# The Discovery of the Application of Ether for the Prevention of Pain

**Published:** 1848-04

**Authors:** 


*The Discovery of the Application of Ether for the Prevention of Pain.*—We received, a few weeks since, a number of Littell's Living Age, containing an elaborate report of the trustees of the Massachusetts General Hospital, of their investigation of the relative claims of Drs. Jackson and Morton to the above discovery; and we had intended giving, in the present number of the Journal, a condensed summary of the conclusions to which they had arrived; but as Dr. Jackson has requested a suspension of public opinion until the publication of a statement which he is engaged in preparing shall be made, we have concluded to defer doing so until we see what he has to say upon the subject.

